# Range expansion and reproduction of the ectoparasitic deer ked (*Lipoptena cervi*) in its novel host, the Arctic reindeer (*Rangifer tarandus tarandus*), in Finland

**DOI:** 10.1007/s00436-020-06817-x

**Published:** 2020-07-23

**Authors:** Sanna-Mari Kynkäänniemi, Raine Kortet, Sauli Laaksonen

**Affiliations:** 1 Department of Ecology and Genetics, University of Oulu, P.O. Box 3000, FI-90014 Oulu, Finland; 2 Department of Environmental and Biological Sciences, University of Eastern Finland, P.O. Box 111, FI-80101 Joensuu, Finland; 3 Department of Veterinary Biosciences, Faculty of Veterinary Medicine, University of Helsinki, P. O. Box 33, FI-00014 Helsinki, Finland

**Keywords:** Adaptation, Deer ked, Range expansion, Reindeer, Reproduction

## Abstract

The deer ked (*Lipoptena cervi*) is a harmful ectoparasite that emerged in the reindeer herding area of Finland in 2006. To understand the current range and the intensity of infestations on its novel reindeer host, we studied deer ked pupae collected from reindeer and moose bedding sites and conducted a questionnaire survey among the managers of 18 reindeer herding cooperatives in the southern part of the reindeer herding area. Our study confirmed that the deer ked can survive and successfully reproduce on reindeer through winter and that flying deer keds had been observed in reindeer wintering areas during several autumns in twelve cooperatives. The pupae originating from reindeer were smaller and showed lower hatching rates than the pupae from moose. The present results indicate that the range of the deer ked infestations on reindeer in Finland expanded during the recent 5 years, now reaching 14 cooperatives and bordering an area south of approximately 66° N 25° E in the west and 65° N 29° E east.

**Electronic supplementary material:**

The online version of this article (10.1007/s00436-020-06817-x) contains supplementary material, which is available to authorized users.

## Introduction

The deer ked was first detected in the south-eastern Finland in the 1960s, presumably spreading from Russia (Kaitala et al. 2009). The adults feed on cervid hosts blood and reproduce in its fur. The viviparous female produces one pre-pupated larva at a time, which drops off the host and new adults emerge next autumn (Härkönen et al. 2012). The distribution of the deer ked population in Finland has widened rapidly, reaching the southern reindeer herding area in the 2000s and the northern limit being approximately at 65° N in 2010 (Välimäki et al. 2010; Jaakola et al. 2015).

The deer ked infests different cervids with various levels of success. Its principal host in Finland is the moose (*Alces alces*) (Kaunisto et al. 2009). There are few earlier observations of the pupae from the bedding sites of the reindeer (*Rangifer tarandus tarandus*) and wild forest reindeer (*R. t. fennicus*) (Kaunisto et al. 2009; Välimäki et al. 2011). Välimäki et al. (2011) reported heavier pupae found in Finnish moose compared with those found in wild forest reindeer and Norwegian roe deer (*Capreolus capreolus*). The deer ked in Sweden produces larger pupae in the roe deer, which also survive better than pupae produced in moose in the same area (Härkönen et al. 2015). Despite the differences in host use, the genetics of the deer ked between the eastern population in Finland and western population in Sweden and Norway seem to be similar (Jaakola et al. 2015). The relationship between the deer ked and its new host, the reindeer, is still poorly studied. When encountering a new host, the parasite needs to overcome physiological and immunological defenses of the host to successfully attach and reproduce (Moore 2002; Wall 2007), but external climate and biotic factors can also have effects on the new host-parasite relationship (Wall 2007).

The first aim of this study was to investigate whether the deer ked can reproduce on the reindeer over winter in natural conditions. The second aim was to explore the current distribution of the deer ked infestation on reindeer using a questionnaire survey. We hypothesized that the hatching rate of the deer ked pupae originating from reindeer would be reasonably low as this relationship has been very recently established (Kynkäänniemi et al. 2010; Välimäki et al. 2011), but that the distribution of the deer ked infestation on reindeer would have been widening over recent years.

## Material and methods

### Pupal data

**Fig. 1:**
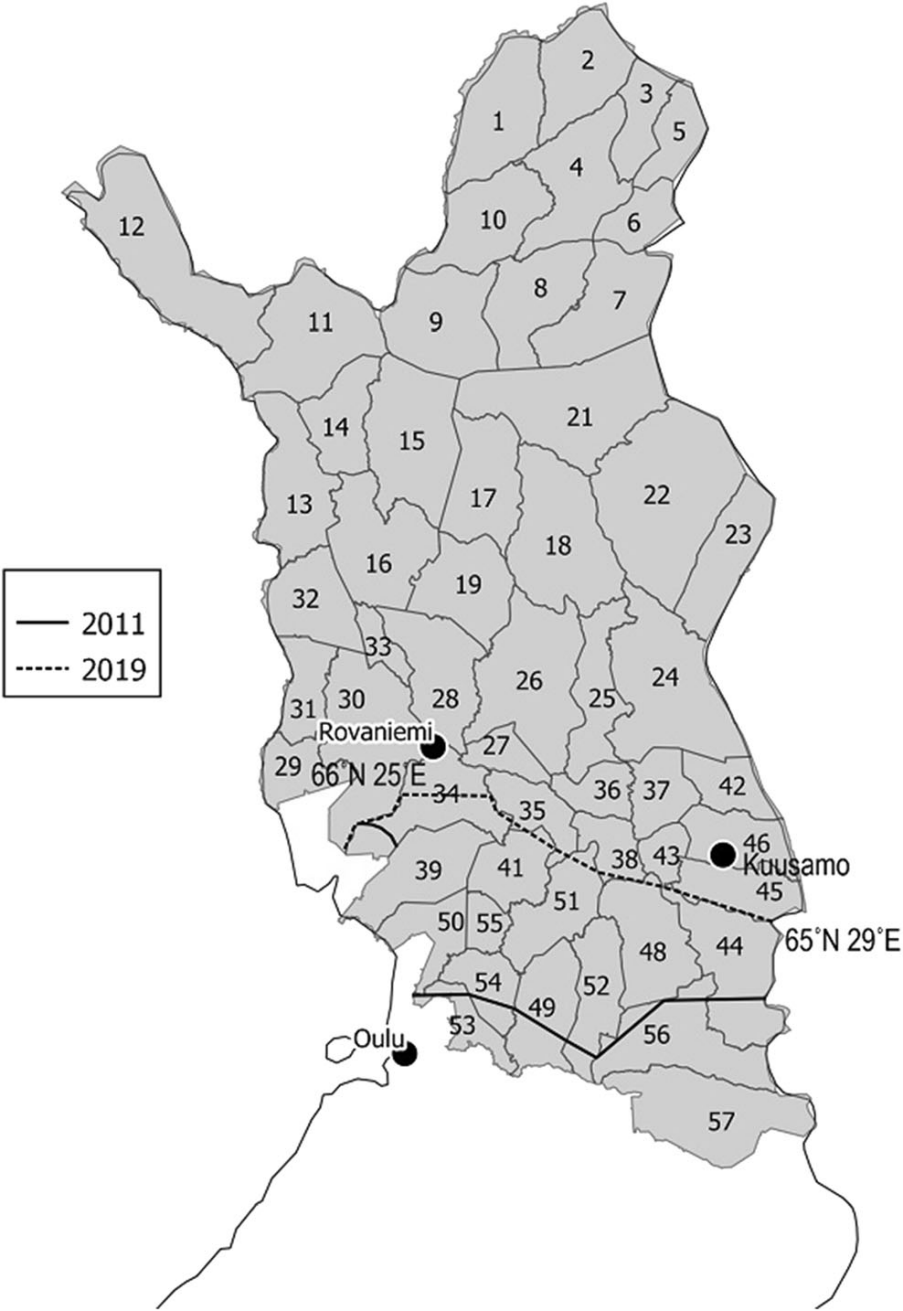
Current range (dashed line) and range in 2011 (solid line) of the deer ked infestation among reindeer in Finland. At present, the distribution range overlaps 66° N 25° E in the west side and 65° N 29° E in the east side of the country. The data is based on the questionnaire survey to the managers of reindeer herding cooperatives. Numbered areas are cooperatives of reindeer herding area (source Reindeer Herders’ Association)

Pupae (*n* = 39, excluding five dead) from eleven reindeer bedding sites were collected in the Halla reindeer herding cooperative (Fig. 1, number code 57; 64° N, 28° E) between January and April of 2011. Pupae were also collected from the bedding sites of six moose (*n* = 19) in the same area in April. The pupae were stored in + 4 °C and transferred to the University of Oulu. The pupae were weighed with a micro balance on 13 May. On 15 May, the pupae were placed in + 17 °C to monitor their hatching success, development time, and adult longevity.

### Questionnaire survey

To support the earlier pupal data and to study the current range of deer ked infestation on reindeer, we performed a questionnaire survey to the managers or representatives of the 18 reindeer herding cooperatives in the southern herding area in Finland. Knowledge on the current range of the deer ked infestation on reindeer would give additional information to understand the relationship between the reindeer and the deer ked. The survey was conducted through phone interviews in September and October 2019. The survey (Online Resource 1) consisted of seven structured questions with options to answer yes/no/do not know and four open questions. Questions related to annual observations of deer ked infested reindeer and infestation-related clinical signs, local range of the deer ked infested reindeer, observations on areas where deer keds fly, pastures of reindeer and moose, and possible practices of antiparasitic treatment. The respondent’s knowledge of the infestation-associated symptoms of reindeer (Kynkäänniemi et al. 2014) was confirmed before the interviews.

### Statistical analyses

We analyzed the pupal data with R (*v.* 3.0.1., R Core Team 2013). To test the differences in pupal mass between the pupae found from reindeer and moose, we used a two-sample *t* test. To analyze the explanatory factors (host; reindeer, moose) for pupal mass, development time, and adult longevity, a linear mixed effect model (package nlme) was used, each as a dependent variable at a time.

## Results and discussion

### Pupal data

The hatching rate of deer keds was 38.5% for the pupae found from reindeer bedding sites and 73.7% for the pupae found from moose bedding sites. Semi-domesticated reindeer and wild forest reindeer (*Rangifer tarandus fennicus*) have previously been reported as auxiliary hosts for deer ked in Finland (Välimäki et al. 2011) and our results confirmed that notable numbers of deer keds can live and reproduce on reindeer until April.

The average pupal masses were 8.63 mg (SD; 1.01, *n* = 39) for reindeer and 8.99 mg (SD; 1.41, *n* = 19) for moose. When only the masses of the hatched pupae were compared, the pupae from reindeer were lighter (8.79 mg, SD; 0.48, *n* = 15) than those from moose (9.59 mg, SD; 0.73, *n* = 14) (*p* = 0.002). Development times were (reindeer: mean; 83.27 days, SD; 4.84, moose: mean; 82.50 days, SD; 5.42) and adult longevities were (reindeer: mean; 57.00 days, SD; 12.01, moose: mean; 60.57 days, SD; 9.69). In the linear mixed effect models (Table 1), the host was a significant explanatory factor for pupal mass. When the development time was set as the dependent variable, it was shortened by heavier pupal mass and adult longevity. Adult longevity was lowered by a longer development time. The results of the linear mixed effect model suggested that heavier pupae from moose had shorter development times and longer adult longevity. Välimäki et al. (2011) reported heavier pupal masses from Finnish moose compared with wild forest reindeer. Heavier pupae have been shown to exhibit longer development times (Härkönen et al. 2013) and pupae are known to be smaller in the northern areas (Kaunisto et al. 2011). According to Härkönen et al. (2013), weight loss during diapause is critical for the survival of pupae through post-diapause development. Interestingly, Kaunisto et al. (2011) reported that small adults hatched earlier from smaller pupae than larger ones and the diapausing pupae were heavier in southern Finland compared with those in northern Central Finland.

**Table 1:** Parameter estimates of linear mixed effect model. Pupal mass, development time, and adult longevity were compared and effect of the host (reindeer/moose) was evaluated

Response	Parameters	Estimate	SE	*t* value	Pr(>|*t*|)
Pupal mass	Intercept	14.742	2.206	6.684	5.28e-07
	Host	0.815	0.210	3.878	0.001
	Development time	− 0.059	0.023	−2.592	0.016
	Adult longevity	− 0.018	0.011	−1.724	0.097
	Multiple R-squared, 0.468				
	Adjusted R-squared, 0.4045				
Development time	Intercept	126.875	13.569	9.351	1.22e-09
	Host	2.841	1.998	1.422	0.168
	Pupal mass	− 3.597	1.388	− 2.592	0.016
	Adult longevity	0.210	0.077	− 2.722	0.0117
	Multiple R-squared, 0.3362				
	Adjusted R-squared, 0.2566				
Adult longevity	Intercept	198.452	52.018	3.815	0.001
	Host	7.339	4.491	1.634	0.115
	Development time	− 1.087	0.399	− 2.722	0.012
	Pupal mass	− 5.793	3.360	− 1.724	0.097
	Multiple R-squared, 0.2638				
	Adjusted R-squared, 0.1755				

### Questionnaire

The questionnaire revealed that the deer ked has parasitized reindeer in 14 reindeer herding cooperatives in the southern herding area (Fig. 1). There were observations of flying deer keds in 14 cooperatives from Autumn 2019. Seventeen of 18 managers of the cooperatives knew how the deer ked–infested reindeer appear and behave. Reindeer generally show strong behavioral responses against deer ked (Kynkäänniemi et al. 2014). There was spatial variation in the ked infestation patterns in three cooperatives over the years. In one cooperative in the north (Fig. 1, number 34), there were 10 years between the first observations from the west and east sides of the cooperative. According to reindeer herders in the western area, the, presumably, warmer weather near the Baltic Sea and River Kemijoki may have enhanced the expansion. In two reindeer herding cooperatives (52 and 54), which are divided by River Iijoki, there were 2 to 5 years of delay in observations of deer ked–infested reindeer between southern and northern sides of the cooperatives. Minor delays were also observed in the two nearby cooperatives (48 and 49).

The first observations of the deer ked–infested reindeer had occurred in 2006, when the invasion of the deer ked became acknowledged (Table 2). The first wave of the deer ked parasitism reached seven reindeer herding cooperatives from 2006 to 2011. Our questionnaire data suggests that there is an ongoing second wave of expansion of the deer ked infestation on reindeer towards the north as the observations increased in seven reindeer herding cooperatives from 2014 to 2019 (Table 2). In 2009, the distribution limit of the deer ked was estimated to be near the border of the southern reindeer herding area in Finland (Kaunisto et al. 2009). According to Välimäki et al. (2010), there were observations on flying deer keds up to 65° N north in Finland in 2010. Our data demonstrates that deer ked–infested reindeer have been observed in only six districts in 2009, but now (in 2019), there are 14 deer ked–infested districts. At present, the northernmost distribution limit of the deer ked–infested reindeer runs from approximately 66° N 25° E in the west to 65° N 29° E on the east side of Finland.

**Table 2:** The expansion of the deer ked parasitism on reindeer. The first observations are from the 13 years back (2006). The results suggest that there might have been a second wave expansion of the deer keds on reindeer towards the north as the observations have increased during the past 5 years

Start point of the deer ked infestation on reindeer (2006–2019)	Numbers of the cooperatives (see Fig. 1. for the ID numbers)
2016–2017	2 (41, 48)
2015–2016	2 (50, 51)
2014–2015	3 (39, 52, 55)
2011	1 (44)
2009	3 (53, 54, 56)
−2006	3 (34, 49, 57)

In 12/14 reindeer herding cooperatives, flying deer keds were noted in Autumn in places where reindeer were supplementary fed during winter. This suggests that deer ked pupae were dropped to the ground from reindeer during the winter and then hatched in the autumn. Seven of 14 cooperatives reported observations of harassing numbers of deer keds in the corrals used for supplementary feeding of reindeer that provided no access to other cervid species. In 14 cooperatives, there was a possibility that the reindeer could have deer keds hatched from pupae born on moose as the overwintering areas of the species overlap. In 9/14 cooperatives, there was a possibility that reindeer can have deer keds hatched from pupae from other reindeers. Our results suggest that both reindeer and moose can have an effect on deer keds’ adaptation to the local environment in the reindeer herding area. In our survey, we did not ask about the properties of flying deer keds, but one respondent noted that the deer keds were darker and harder 7 years ago. Continuous weighing of the hatched offspring would provide information on the possible future adaptation processes between reindeer and the deer ked. According to Härkönen et al. (2015), the body size of the deer ked is bigger in Finland than in Sweden and Norway, where it parasitizes several cervid host species. Further research on the reproduction success and interaction between the deer ked, reindeer, and the surrounding moose population is needed, as the pastures of the moose and reindeer are partially overlapped, and ongoing climate change likely promotes the future spread of this invasive ectoparasite. In the circumpolar area, the ambient temperature has been predicted to increase and likely enhance the establishment of ectothermic species like parasitic flies.

## Electronic supplementary material

ESM 1 (DOCX 19 kb)
