# Supplementary material for: Range expansion and reproduction of the ectoparasitic deer ked (Lipoptena cervi) in its novel host, the Arctic reindeer (Rangifer tarandus tarandus), in Finland
Source: Parasitol Res. 2020 Jul 23;119(9):3113–7. doi: 10.1007/s00436-020-06817-x (PMC7431400; doi:10.1007/s00436-020-06817-x)
Supplement: Supplementary file 1 — (DOCX 19 kb) [file 436_2020_6817_MOESM1_ESM.docx]

PARASITOLOGY RESEARCH, Appendix 1; Online Resource 1

**Range expansion and reproduction of the ectoparasitic Deer ked (*Lipoptena cervi*) in its novel host, the Arctic reindeer (*Rangifer tarandus tarandus*)**

Sanna-Mari Kynkäänniemi^1*^, Raine Kortet^2^, Sauli Laaksonen^3^

^1^University of Oulu, Department of Biology, P.O. Box 3000, FI-90014 Oulu, Finland

^2^University of Eastern Finland, Department of Environmental and Biological Sciences, P.O. Box 111, FI-80101 Joensuu, Finland

^3^University of Helsinki, Department of Veterinary Biosciences, Faculty of Veterinary Medicine, P. O. Box 33, FI-00014 Helsinki, Finland

*author for correspondence, e-mail: sanna-mari.kynkaanniemi@oulu.fi

Questionnaire survey conducted among the managers of 18 reindeer herding cooperatives in the southern part of the reindeer herding area. The survey consisted of seven structured questions with options to answer yes/no/don’t know and four open questions (see below).

QUESTIONS:

1a. Have you observed flying deer keds in the area of the herding cooperative in 2018?

1b. Have you observed flying deer keds in the area of the herding cooperative in 2019?

(*yes/no/don’t know*)

2. Do you know how the deer-ked-infested reindeer appear and how they behave? (*yes/no*)

3. Have you observed deer-ked-infested reindeer in the herding cooperative in 2017-2019? (*yes/no/don’t know*)

4. Since when have you observed deer-ked-infested reindeer in your herding cooperative? (open)

5. Are there areal within-cooperative differences in the occurrence of the deer-ked-infested reindeer? (open)

6. What are the practices for antiparasite treatments in the herding cooperative? (open)

7. Do the pastures of the reindeer and moose overlap in the herding cooperative? (*yes/no/don’t know*)

8. The deer ked flies from the late summer to the early Autumn. Is there any possibility that deer ked pupae would have dropped from reindeer in the area where the reindeer are during the flight season of the deer ked? (*yes/no/don’t know*)

9. Is there any possibility that reindeer would move or feed in moose’s winter pasture areas during the deer ked’s flyingseason ? (*yes/no/don’t know*)

10. If the deer ked pupae drops from the reindeer during winter, do the moose move near these areas when deer keds fly? (*yes/no/don’t know*)

11. Have you observed flying deer keds in the reindeer winter corral areas in Autumn? (open)
